# Pharmacological Dopamine Manipulation Does Not Alter Reward-Based Improvements in Memory Retention during a Visuomotor Adaptation Task

**DOI:** 10.1523/ENEURO.0453-17.2018

**Published:** 2018-07-16

**Authors:** Graziella Quattrocchi, Jessica Monaco, Andy Ho, Friederike Irmen, Wolfgang Strube, Diane Ruge, Sven Bestmann, Joseph M. Galea

**Affiliations:** 1 Sobell Department of Motor Neuroscience and Movement Disorders, UCL Institute of Neurology, University College London, London WC1N 3BG, United Kingdom; 2 Brain Connectivity Centre, C. Mondino National Neurological Institute, Pavia I-27100, Italy; 3 Department of Psychiatry and Psychotherapy, Ludwig-Maximilians-University LMU, Munich D-80336, Germany; 4 Department of Psychology and Neurosciences, Leibniz Research Centre for Working Environment and Human Factors, Technical University Dortmund, Dortmund D-44139, Germany; 5 School of Psychology, University of Birmingham, Birmingham B15 2TT, United Kingdom

**Keywords:** adaptation, motor learning, punishment, reward

## Abstract

Motor adaptation tasks investigate our ability to adjust motor behaviors to an ever-changing and unpredictable world. Previous work has shown that punishment-based feedback delivered during a visuomotor adaptation task enhances error-reduction, whereas reward increases memory retention. While the neural underpinnings of the influence of punishment on the adaptation phase remain unclear, reward has been hypothesized to increase retention through dopaminergic mechanisms. We directly tested this hypothesis through pharmacological manipulation of the dopaminergic system. A total of 96 young healthy human participants were tested in a placebo-controlled double-blind between-subjects design in which they adapted to a 40° visuomotor rotation under reward or punishment conditions. We confirmed previous evidence that reward enhances retention, but the dopamine (DA) precursor levodopa (LD) or the DA antagonist haloperidol failed to influence performance. We reason that such a negative result could be due to experimental limitations or it may suggest that the effect of reward on motor memory retention is not driven by dopaminergic processes. This provides further insight regarding the role of motivational feedback in optimizing motor learning, and the basis for further decomposing the effect of reward on the subprocesses known to underlie motor adaptation paradigms.

## Significance Statement

Motor adaptation tasks investigate our ability to rapidly adjust motor behaviors. However, these adjustments are transient and the learnt behavior is quickly forgotten. Previous work has found that reward-based feedback can enhance the retention of a newly acquired motor behavior and hypothesized that this effect was dependent on dopamine (DA). Here, we confirmed that reward increases the retention of motor memories but found that this was not influenced by drugs that altered DA availability in the brain. Therefore, these findings fail to confirm a role for DA during reward-based improvements in motor retention and highlight possible limitations of using dopaminergic stimulation to optimize motor memory retention in health and disease.

## Introduction

Motor adaptation tasks have traditionally been considered as investigating an exclusively implicit mechanism, driven by sensory prediction errors (Tseng et al., 2007) and unaffected by motivational feedback (Mazzoni and Krakauer, 2006). Contrary to this assumption, the beneficial effects of reward and punishment during motor adaptation paradigms have been shown (Shmuelof et al., 2012, Galea et al., 2015; Nikooyan and Ahmed, 2015; Gajda et al., 2016; Song and Smiley-Oyen, 2017). Specifically, by using reward- or punishment-based monetary feedback, it was previously shown that the latter accelerated error reduction, while the former increased retention (Galea et al., 2015), findings that have been, at least partially, recently replicated (Song and Smiley-Oyen, 2017). These results point toward the existence of independent mechanisms underpinning learning and retention, but also toward differential neural processes driving the effects of reward and punishment during motor adaptation tasks.

The reward system relies heavily on dopamine (DA), with DA neurons firing in response to reward and reward predictors (Volman et al., 2013; Schultz, 2016). In rodents, dopaminergic projections to the motor cortex (M1) are required for successful motor skill learning, and in particular for long-lasting storage of motor memories (Molina-Luna et al., 2009; Hosp, et al., 2011, 2013). These projections originate mainly from the rostro-lateral ventral tegmental area (VTA) and the rostro-medial portion of the substantia nigra, and thus form part of the reward meso-cortico-limbic system (Hosp et al., 2011). Based on this work, it has been hypothesized that reward may improve motor memory retention by promoting plastic changes in M1 through the release of DA (Hosp and Luft, 2013). In addition, administration of levodopa (LD), a precursor of DA, improves motor learning in elderly healthy adults (Flöel, et al., 2005a, 2008a, 2008b) and stroke patients (Flöel, et al., 2005b; Rösser et al., 2008). Indeed, dopaminergic stimulation coupled with motor rehabilitation has been proposed as a possible tool for improving motor recovery after stroke (Scheidtmann et al., 2001).

While DA is important to learn from rewards, its role in mediating the effect of punishment on adaptation is unclear. Indeed, the “single-dimension” hypothesis proposes that DA (but also any other reward-sensitive circuits) is also sensitive to punishment (Wang and Tsien, 2011), whereas the “two-dimension” hypothesis suggests that some dopaminergic neurons are sensitive only to reward, and others only to punishment (Mirenowicz and Schultz, 1996; Matsumoto and Hikosaka, 2009; Fiorillo, 2013). Moreover, another neuromodulator, namely serotonin, has been associated with the anticipation and/or the delivery of punishment (Deakin and Graeff, 1991; Amo et al., 2014; Dayan and Huys, 2015), thus making the study of punishment-related effects even more complex.

A deeper understanding of the neural mechanisms underpinning the effect of reward and punishment during motor adaptation tasks could inform attempts to potentiate the beneficial impact of motivational feedback on motor learning in health and in clinical rehabilitation. Indeed, the need to target motor recovery at multiple sites along the motor learning network by combining motor robotic therapy with pharmacotherapy and reward learning has already been pointed out (Tran et al., 2016).

We sought to investigate the role of DA during a motor adaptation task under reward or punishment conditions. To this end, we tested young healthy participants in the presence of reward- or punishment-based monetary feedback. In a placebo-controlled double-blind design, we examined the role of DA by either increasing DA availability with LD (DA precursor) or decreasing DA effects with haloperidol (DA antagonist). We predicted that manipulating the dopaminergic system would specifically alter the impact of reward-based feedback on motor memory retention.

## Materials and Methods

### Participants

A total of 96 participants [age 18–40 years, 23.34 ± 4.39 years (mean ± SD), *n* = 60 females] was recruited from the University College London Psychology pool who fulfilled the following criteria: (1) right-handed (as assessed with the Edinburgh handedness inventory; Oldfield, 1971); (2) 18–45 years old; (3) no self-reported history of major medical disorders or drug abuse; (4) normal or corrected-to-normal vision; (5) no drug allergies; (6) currently taking no medication that would affect the central nervous system or interfere with the absorption of LD; and (7) not pregnant (self-report). The suitability of the participants for the pharmacological protocol was evaluated based on a review of their clinical history by a medical doctor. All participants were naïve to the experimental aims and provided written informed consent. The experiment was approved by the University Research Ethics Committee and was conducted in accordance with the principles expressed in the Declaration of Helsinki.

### Cognitive scales

All participants underwent a battery of validated neuropsychological tests. The mini-mental state examination (Folstein et al., 1975) was used as a general cognitive screening tool, while the frontal assessment battery (Dubois et al., 2000) and the Stroop test (Stroop, 1935) assessed executive functions. We also evaluated apathy (apathy evaluation scale; Marin et al., 1991), depression (Beck depression inventory; Beck et al., 1961), and sensitivity to punishment and reward (SPSRQ-20; Aluja and Blanch, 2011). To control for the effect of sleep, participants were asked to sleep at least 6.5 h the night before the study day (Al-Sharman and Siengsukon, 2013). After completion of the session, participants reported whether they thought they had taken the active drug or placebo and scored their levels of alertness on a 10-point visual analog scale (0 = very sleepy, 10 = fully alert). All this information allowed us to control for trait and state differences across groups.

### Experimental task

We used a standard visuomotor adaptation reaching task (Krakauer et al., 2000; Taylor and Ivry, 2014). Participants sat with their forehead supported in front of a workstation while holding the handle of a two-joint robotic manipulandum with their dominant right arm. The forearm was stabilized by straps to a molded cast. A horizontal mirror, suspended 2 cm above the hand, prevented direct vision of the arm, but showed a reflection of a screen mounted above. Online visual feedback regarding hand position was provided by a white cursor (0.3 cm in diameter) projected onto the screen. In some blocks, the online visual feedback of the cursor was removed (no vision).

**Figure 1.**
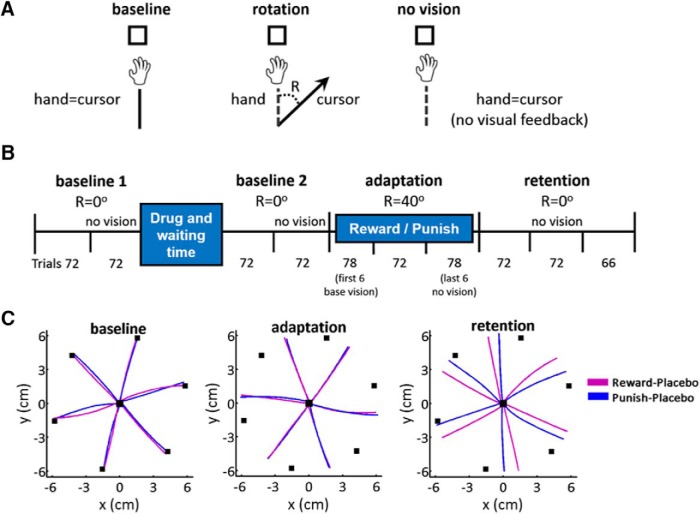
Task and paradigm. ***A***, Task. Participants made 6 cm reaching movements to a target. Visual feedback was perturbed by a 40° clockwise rotation (R) in adaptation phase (rotation). In no vision trials, the cursor and the hand position corresponded but there was no visual feedback. ***B***, Study protocol. Participants completed 72 trials of baseline training with veridical visual feedback, followed by 72 baseline trails with no visual feedback (no vision). Drug (LD/haloperidol/placebo) was then administered and participants waited the corresponding waiting time (1 h for LD or placebo, 2 h for haloperidol). After that, the two baseline blocks were repeated (baseline 2). During adaptation, visual feedback was perturbed 40° clockwise for 216 trials (three blocks). To avoid this starting abruptly at the beginning of a block, the first adaptation block started with six baseline trials with veridical visual feedback, followed by 72 trials with the perturbation. Then, participants were exposed to 216 (retention, three blocks) trials with no perturbation and no visual feedback. Again, to avoid a context change at the beginning of a block, the last adaptation block finished with six retention trials (i.e., total 78 trials in last adaptation block, followed by two retention blocks of 72 trials and one block of 66 trials). ***C***, Hand trajectories toward each target of one representative subject in the R-Pl (violet) and punish-placebo (blue) group. From left to right, Last trial toward each target of baseline 1, last trial toward each target of adaptation, last trial toward each target of retention.

The task consisted of center-out fast ballistic movements to visual targets. Participants had to initially bring the cursor within a 1 cm^2^ starting box located in front of the body’s midline. Once the cursor was within the starting point, a white 0.5 cm^2^ target appeared pseudo-randomly in one of six positions arrayed radially at 6 cm from the start (15°, 75°, 135°, 195°, 255°, and 315° clockwise, with 0° representing 12 on a clock). Participants were instructed that, when ready, they should make a fast, accurate, “shooting” movement through the target, avoiding corrections. As the cursor crossed an imaginary 6-cm radius circle centered at the starting position, a green dot appeared at the endpoint. After 500 ms, the manipulandum returned the hand back to the start. Participants were instructed that they had to try to maintain a constant and relatively fast speed across the whole experiment. To encourage this, the target turned red or blue if the movement duration was >300 or <100 ms, respectively. This time criteria was just used as feedback, but trials were not removed based on this time (see below). In the adaptation trials, the manipulandum introduced a visuomotor perturbation, in which the cursor position was rotated 40° clockwise from the actual hand position (Fig. 1*A*,*C*).

### Reward and punishment feedback

During the adaptation phase, the reward groups accumulated positive points and the punishment groups accumulated negative points. Points were calculated based on angular endpoint error, i.e., the difference between the cursor endpoint angle and the target angle, as follows:

Reward: 4 points: < 1°; 3 points: 1 − 5°; 2 points: 5 − 15°; 1 point: 15 − 25°; 0 points: ≥25°.

Punishment: 0 points: < 1°; −1 point: 1 − 5°; −2 points: 5 − 15°; −3 points: 15–25° degrees error; −4 points: ≥25°.

Both the points received on a trial-by-trial basis and the cumulative score of the block were shown. Participants were informed that points had a monetary value (3.47 pence/point) and depended on performance. Participants in the reward groups started with £0 and could earn up to £30 based on the accumulated points, while those in the punishment groups were given an initial amount of £30 and lost money based on the cumulative negative points.

### Experimental protocol

The study was composed of four phases (Fig. 1*B*). Participants initially performed a baseline (baseline 1) composed of one block (72 trials) with visual feedback and one with no visual feedback (no vision) of the cursor (nor of the endpoint green dot). After the drug/placebo administration and the waiting time, a second equivalent baseline (baseline 2) was performed. The cursor was then rotated 40° clockwise and reward/punishment feedback was provided as described above for three blocks (adaptation). To avoid the perturbation beginning at the start of a block, the first adaptation block started with six baseline trials with veridical visual feedback and no reward/punishment feedback, followed by 72 trials with the perturbation. Finally, participants were exposed to 216 (retention, three blocks) trials with no perturbation and no visual feedback (retention). Again, to avoid this change in context starting at the beginning of a block, the last adaptation block finished with six retention trials (i.e., there were 78 trials in the last adaptation block, followed by two retention blocks of 72 trials and 66 trials). The removal of visual feedback of the cursor restricts re-learning and therefore the observed gradual drift back to baseline performance represents memory retention (Galea et al., 2011; Kitago et al., 2013;). Each block was separated by a short (<1 min) rest period.

### Randomization and blinding procedure

Participants were randomly allocated to one of six groups (*n* = 16 per group): reward-LD (R-LD), punishment-LD (P-LD), reward-haloperidol (R-Halo), punishment-haloperidol (P-Halo), reward-placebo (R-Pl), and punishment-placebo (P-Pl). After baseline 1, subjects received either 100 mg of the DA precursor LD (plus 25 mg of carbidopa) or 2.5 mg of the D1/D2-antagonist haloperidol or placebo. We used a nonselective DA-receptor antagonist as motor learning depends on both D1- and D2- receptors mechanisms (Molina-Luna et al., 2009), probably through the activation of the intracellular phospholipase-C pathway in M1 (Rioult-Pedotti et al., 2015). To coincide with the peak plasma concentration of LD (Nutt and Fellman, 1984) and haloperidol (Tomassini et al., 2016), the task was restarted, respectively, after a 60-min wait for LD and placebo groups and a 120-min wait for Halo groups. During the waiting period participants sat quietly in the laboratory. The randomization and administration of the drug were performed by a medical doctor, whereas the examiner and participants were naïve to the aim of the experiment and blinded to the drug/placebo status. All participants were told that they will receive either a placebo tablet or an active drug (LD or placebo). The doses and administration times were similar to previous studies that have shown clear behavioral and neurophysiological effects for LD and haloperidol (Bestmann et al., 2015). All participants fasted for at least 2 h preceding drug/placebo intake to prevent interference with drug absorption (Nutt and Fellman, 1984). No adverse events were reported.

### Data analyses

The 2D (*x*, *y*) position of the hand was collected through a custom C++ code at a sampling rate of 100 Hz. Movement onset was defined as the point at which radial velocity crossed 10% of peak velocity. Movements were considered terminated when the cursor breached the 6-cm target perimeter. Performance was quantified using angular reach direction (AD, ^o^), i.e., the difference between the target angle and the angular hand position at the end of the movement (Hadipour-Niktarash et al., 2007). During veridical feedback, the goal was for reach direction to be 0°. With the visuomotor perturbation, reach direction had to compensate; i.e., for a +40° (clockwise) visuomotor rotation, a reach direction of -40° (counter-clockwise) was required. To adjust for between-subject baseline directional biases (Ghilardi et al., 1995), AD was corrected by subtracting the average AD of the first baseline one block from the trials with cursor vision, and the average AD of the second baseline one block (“no vision”) to the trials with no visual feedback of the cursor (Krakauer et al., 2005).

Reaction time (RT; time between target appearance and movement onset) and movement time (MT; time between movement onset and movement end) were calculated for each trial. Trials in which AD exceeded 20° or was less than -60° (Tanaka et al., 2009; Galea et al., 2015), or MT or RT exceeded 1000 ms or were <100 ms, were removed. This accounted for 1.67% of trials. Epochs of all kinematics were created by averaging across 6 consecutive trials (Krakauer et al., 2005; Galea et al., 2011). For the purpose of analysis, the first six trials of the first adaptation block (which were still without perturbation, as described in Experimental protocol) were annexed to baseline 2, while the final six trials of the last adaptation block (without vision and no perturbation, see Experimental protocol) were considered as retention.

Data and statistical analysis were performed using MATLAB (version R2013a, The MathWorks) and IBM SPSS (version 21.0). Differences between demographics, cognitive scores, baseline MT, RT, and AD were evaluated by separate one-way ANOVAs (quantitative data) or χ^2^ or Fisher’s exact test (proportions).

We first performed repeated-measure ANOVAs for each study phase (adaptation, retention) by comparing AD with drug (placebo*LD*haloperidol) and feedback (reward*punishment) as between-subject factors, and blocks as a within-subject factor (three blocks in adaptation, three blocks in retention).

A model-based analysis was also performed. Specifically, we applied a single-rate state-space model (SSM; Thoroughman and Shadmehr, 2000; Donchin et al., 2003; Tanaka et al., 2009; Galea et al., 2015) to each participant’s entire dataset. This has the advantage of estimating learning and retention rates from all available data, with no arbitrary selection of time points or trials of interest. The SSM took the following form:

$$y_{n}=-z_{n}^{t}$$

$$z_{n+1}^{t}=Az_{n}^{t}+B(r_{n}-z_{n}^{t})$$

$y_{n}$ represents the angular direction (relative to target) on trial *n*; $z_{n}^{t}$ is the state of the learner, i.e., the current estimated visuomotor mapping (rotation) with the target *t*; $r_{n}$ represents the visuomotor rotation that was imposed on trial *n;* $r_{n}-z_{n}^{t}$ is the error in the visuomotor mapping (i.e., cursor error). The learning rate (B) determines how much of the cursor error $\left(r_{n}-z_{n}^{t}\right)$ is adapted for. In addition, the visuomotor mapping slowly forgets at a rate determined by the scalar parameter A (decay rate). During blocks with no visual feedback (no vision, retention phase) we assume that B = 0. Therefore, in this case, the system forgets with constant A (with larger values signifying increased retention). Using the MATLAB function fmincon, for each subject we estimated A and B to minimize the squared error between trial-by-trial predicted hand direction ($y_{t(n)}$) and actual trial-by-trial hand direction, subject to constraints (0 < A < 1) and (-1 < B < 1). The model’s goodness of fit was determined using R^2^. As the assumption of normality was violated, we examined between-groups differences for the A and B parameters using an adjusted rank transform (ART) test (Leys and Schumann, 2010; Chan, 2014), with feedback (reward*punishment) and drugs (placebo*LD*haloperidol) as independent variables.

All data were tested for normality using the Shapiro–Wilk test and nonparametric tests were used when warranted (as indicated in the tables and text). Homogeneity of variance was evaluated using Levene test and Welch test was used when this assumption was violated. Greenhouse–Geisser (if epsilon, ε < 0.75) or Huynh–Feldt (if ε > 0.75) corrections were used when sphericity was violated (Mauchly’s test). Tukey *post hoc* test was used when warranted. No statistical methods were used to predetermine sample sizes, but our sample sizes are similar to those reported in previous studies (Galea et al., 2015, 2011). Significance level was set at *p* < 0.05. Effect sizes were provided by phi for χ^2^ test, Cohen’s *d* for *t* tests or *r* score for Mann–Whitney test, partial η (η^2^) for ANOVA, and ε^2^ for Kruskal–Wallis *H* test.

## Results

### Demographics, cognitive and kinematic parameters were similar across groups

We investigated the effect of LD or haloperidol on a motor adaptation task under reward or punishment in six groups (*n* = 16 each): R-LD (age 19–40 years, 23.4 ± 5 years, *n* = 8 females), P-LD (age 18–28, 22.4 ± 2.8, *n* = 10 females), R-Halo (*n* = 16, age 21–39 years, 26.1 ± 5, *n* = 13 females), P-Halo (*n* = 16, age 19–37, 23.1 ± 4.6, *n* = 9 females), R-Pl (age 20–40, 25 ± 4.7, *n* = 10 females), and P-Pl (age 19–28, 22.5 ± 2.2, *n* = 10 females). As shown in Table 1, all groups were comparable for body mass index, education level, cognitive scores, amount of money received at the end of the session, and success rate, defined as number of times they received the maximum points (i.e., four points in the reward groups and zero points in the punishment groups). Participants’ alertness at the end of the session was similar across groups [R-LD = 7.6 ± 0.3, mean ± SEM, P-LD = 7.1 ± 0.3, R-Halo = 7.1 ± 0.6, P-Halo = 5.9 ± 1.3, R-Pl = 7.2 ± 0.4, P-Pl = 7 ± 0.2; *F*_(5,90)_ = 2.2, *p* = 0.058, η^2^ = 0.110]. Thirteen of the 32 (41%) participants in the placebo groups believed they had received LD, whereas 18 of 32 (56%) in the LD groups and 19 of the 32 in the haloperidol groups (59.4%) believed they had received placebo, thus showing that the blinding protocol was effective.

**Table 1.** Participants’ characteristics

	R-LD	P-LD	R-Halo	P-Halo	R-Pl	P-Pl	*λ^*2*^_*(5)*_* or *F*_(5,90)_	*p* value	Effect size
BMI	21.9 ± 0.7	23.4 ± 0.9	23.5 ± 1.4	21.8 ± 1.1	22.3 ± 0.7	21.5 ± 0.5	0.835	0.528	0.046
Education	13 (81.3)	11 (68.7)	15 (93.8)	13 (81.3)	15 (93.7)	12 (75)	16.25	0.246	0.024
MMSE	29.7 ± 0.1	29.4 ± 0.3	29.4 ± 0.3	29.7 ± 0.1	29.5 ± 0.2	29.7 ± 0.1	0.60	0.699	0.032
FAB	17.6 ± 0.2	17.6 ± 0.1	17.1 ± 0.4	17.6 ± 0.1	17.6 ± 0.1	17.7 ± 0.1	0.89	0.495	0.047
Stroop E	0.4 ± 0.2	0.7 ± 0.3	0.5 ± 0.2	1.5 ± 0.5	0.4 ± 0.2	1 ± 0.3	1.87	0.109	0.099
Stroop T	4.6 ± 0.9	4.2 ± 1.3	3.9 ± 0.9	5.5 ± 1.7	5.5 ± 0.7	5 ± 2.1	0.27	0.928	0.016
AES-S	28.4 ± 1.3	26 ± 1.5	27.9 ± 1.5	31 ± 1.6	28.7 ± 1.5	30 ± 1.6	1.43	0.221	0.074
BDI	3.3 ± 0.9	2.9 ± 1	3.3 ± 1.1	6 ± 1.7	3.7 ± 1.1	5.2 ± 1.3	1.01	0.422	0.053
SP	3.7 ± 0.7	3.4 ± 0.5	4.8 ± 0.6	4.1 ± 0.6	3.8 ± 0.7	3.7 ± 0.7	0.56	0.732	0.030
SR	4.8 ± 0.6	3.7 ± 0.5	5.2 ± 0.6	5.3 ± 0.5	4.4 ± 0.6	3.8 ± 0.7	1.44	0.218	0.074
Money	18.3 ± 0.4	18.1 ± 0.6	17.7 ± 0.6	18 ± 0.2	18 ± 0.4	18.9 ± 0.3	0.71	0.619	0.038
Success rate	435 (12.6)	454 (13.1)	419 (12.1)	407 (11.8)	454 (13.1)	463 (13.4)	6.44	0.266	0.129

Categorical values are indicated as number and percentages (%), numeric values as mean ± SEM. Comparison between proportions is made with χ^2^ test, comparison between means with one-way ANOVA or Kruskal–Wallis (MMSE, FAB, Stroop, BDI) test. Effect size is provided as phi for χ^2^ test, partial η for ANOVA, and ε^2^ for Kruskal–Wallis. R-LD, *n* = 16; P-LD, *n* = 16; R-Halo, *n* = 16; P-Halo, *n* = 16; R-Pl, *n* = 16; P-Pl, *n* = 16; Education, participants with ≥15 years of education; BMI, body mass index (kg/m^2^); MMSE, mini-mental state examination; FAB, frontal assessment battery; AES-S, apathy evaluation scale, self-administered version; BDI, Beck depression inventory; SP, sensitivity to punishment; SR, sensitivity to reward; Money, GBP (£) received at the end of the session; Success rate, number of trials in which the maximum amount of points was received (i.e., four points in the reward groups and zero points in the punishment groups).

**Figure 2.**
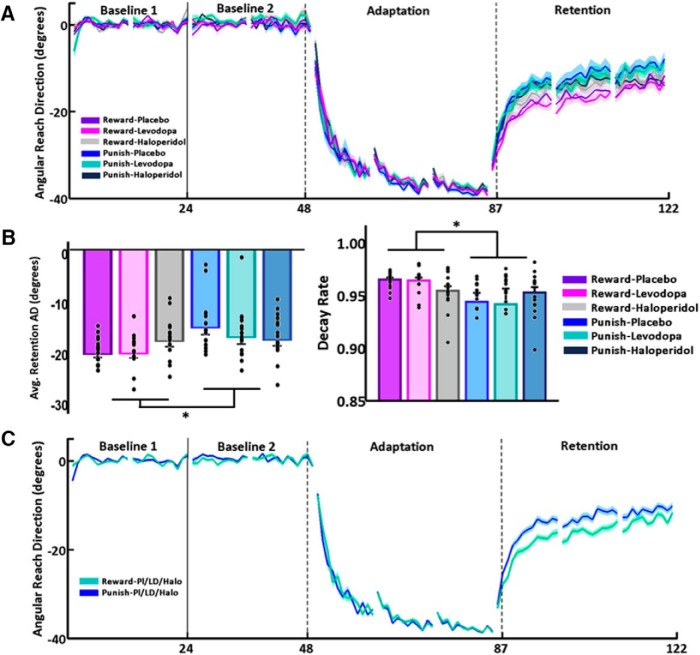
Reward was associated with greater retention than punishment, independently of LD, haloperidol or placebo. ***A***, Epoch (average across six trials) AD (°) during baseline, adaptation, and retention for the six groups (*n* = 16 each). The *x*-axis indicates the number of epochs. The plots represent mean ± SEM. The solid vertical line indicates the wait period after the administration of drug or placebo. The dashed vertical lines indicate the actual beginning and end of first and last adaptation blocks (i.e., the first adaptation block started with six baseline “vision” trials, and the last adaptation block finished with six retention no vision trials). ***B***, Bar graph on the left: average (±SEM) AD (°) for each group during the retention phase. Black dots represent average AD for each participant. The reward groups retained significantly more than the punishment groups [*F*_(1,90)_ = 9.8, *p* = 0.002, η^2^ = 0.098] irrespective of drug status. Bar graph on the right: model parameter A (decay rate, higher values signifying larger retention, average ± SEM) across groups [ART test, *F*_(1,90)_ = 5.51, *p* = 0.021, η^2^ = 0.058]. Black dots represent average decay rate for each participant; *p < 0.05. ***C***, Epoch (average across six trials) AD (°) during baseline, adaptation, and retention for the combined reward groups (*n* = 48) versus the combined punishment groups (*n* = 48).

AD was similar across groups during baseline 1 and baseline 2 (Table 2; Fig. 2*A*). Apart from the R-Pl group showing slower RTs than the punish-placebo group during baseline 2 (*p* = 0.017, Tukey *post hoc* test), MTs and RTs were similar across groups for baseline 1 and 2 (Table 2).

**Table 2.** RTs, MTs, and baseline AD across groups

	R-LD	P-LD	R-Halo	P-Halo	R-Pl	P-Pl	ANOVA
Baseline 1							
RT	317 ± 14	333 ± 17	352 ± 10	350 ± 11	387 ± 29	333 ± 13	*F*_(5,90)_ = 2.01, *p* = 0.085, η^2^ = 0.100
MT	286 ± 14	261 ± 10	303 ± 6	269 ± 8	278 ± 13	278 ± 11	*F*_(5,90)_ = 1.76, *p* = 0.129, η^2^ = 0.089
AD	-0.7 ± 0.3	-1.5 ± 0.5	-0.9 ± 0.4	-1.2 ± 0.3	-1.2 ± 0.5	-0.9 ± 0.2	*F*_(5,90)_ = 0.64, *p* = 0.670, η^2^ = 0.034
Baseline 2							
RT	305 ± 22	318 ± 18	346 ± 9	353 ± 10	374 ± 30	287 ± 14	***F*_**(5,90)**_ = 3.07, *p* = 0.013, η^**2**^ = 0.146**
MT	249 ± 8	259 ± 14	269 ± 7	269 ± 6	255 ± 11	237 ± 7	*F*_(5,90)_ = 0.71, *p* = 0.140, η^2^ = 0.087
AD	-0.7 ± 0.3	-0.4 ± 0.5	-0.3 ± 0.5	-0.9 ± 0.3	-1.2 ± 0.2	-0.9 ± 0.3	*F*_(5,90)_ = 0.78, *p* = 0.569, η^2^ = 0.041
Adaptation							
RT	298 ± 21	327 ± 17	343 ± 8	368 ± 15	371 ± 25	321 ± 23	Fb : *F*_(1,90)_ = 0.005, *p* = 0.941, η^2^ = 0.0 D : *F_*(*_*_2,90)_ = 2.74, *p* = 0.070, η^2^ = 0.06 Fb*D : *F*_(2,90)_ = 2.69, *p* = 0.073, η^2^ = 0.06
MT	243 ± 6	264 ± 15	277 ± 11	279 ± 7	270 ± 15	264 ± 13	Fb : *F*_(1,90)_ = 0.32, *p* = 0.573, η^2^ = 0.004 D : *F*_(2,90)_ = 2.23, *p* = 0.114, η^2^ = 0.05 Fb*D : *F*_(2,90)_ = 0.69, *p* = 0.502, η^2^ = 0.01
Retention							
RT	292 ± 24	288 ± 14	351 ± 8	348 ± 10	338 ± 23	289 ± 12	Fb : *F*_(1,90)_ = 2.01, *p* = 0.160, η^2^ = 0.02 **D : *F*_**(2,90)**_ = 6.78, *p* = 0.002, η^**2**^ = 0.13** Fb*D : *F*_(2,90)_ = 1.31, *p* = 0.276, η^2^ = 0.03
MT	227 ± 8	232 ± 14	262 ± 7	250 ± 6	245 ± 10	231 ± 9	Fb : *F*_(1,90)_ = 0.87, *p* = 0.353, η^2^ = 0.01 **D : *F*_**(2,90)**_ = 3.92, *p* = 0.023, η^**2**^ = 0.08** Fb*D : *F*_(2,90)_ = 0.59, *p* = 0.554, η^2^ = 0.01

Values depict the mean ± SEM by averaging over consecutive epochs for each participant and group. A one-way ANOVA was used to compare mean values across groups during baseline 1 and baseline 2. A multifactorial ANOVA was used to compare mean values across groups, with feedback (reward*punishment) and drug (LD*haloperidol*placebo) as between-groups factors. R-LD, *n* = 16; P-LD, *n* = 16; R-Halo, *n* = 16; P-Halo, *n* = 16; R-Pl, *n* = 16; P-Pl, *n* = 16; RT, in ms; MT, in ms; AD, °; Fb, feedback; D, drug. Significant results are bold.

An ANOVA comparing RTs across groups in the adaptation phase, considering feedback*drug as factors, showed no main effect of feedback [*F*_(1,90)_ = 0.005, *p* = 0.941, η^2^ = 0.00] or drug [*F*_(2,90)_ = 2.74, *p* = 0.070, η^2^ = 0.06], and no significant feedback*drug interaction [*F*_(2,90)_ = 2.69, *p* = 0.073, η^2^ = 0.06]. For RTs in the retention phase there was no effect of feedback [*F*_(1,90)_ = 2.01, *p* = 0.160, η^2^ = 0.02], and no feedback*drug interaction [*F*_(2,90)_ = 1.31, *p* = 0.276, η^2^ = 0.03]. However, there was a main effect of drug [*F*_(2,90)_ = 6.78, *p* = 0.002, η^2^ = 0.13], which was mainly driven by significantly slower RTs in the haloperidol groups compared to the LD groups [*p* = 0.001, Tukey *post hoc* test].

MTs in the adaptation phase showed no main effect of feedback [*F*_(1,90)_ = 0.32, *p* = 0.573, η^2^ = 0.004] or drug [*F*_(2,90)_ = 2.23, *p* = 0.114, η^2^ = 0.05], and no significant feedback*drug interaction [*F*_(2,90)_ = 0.69, *p* = 0.502, η^2^ = 0.01]. Regarding MTs during retention phase, there was no effect of feedback [*F*_(1,90)_ = 0.87, *p* = 0.353, η^2^ = 0.01] and no significant feedback*drug interaction [*F*_(2,90)_ = 0.59, *p* = 0.554, η^2^ = 0.01]. However, there was a main effect of drug [*F*_(2,90)_ = 3.92, *p* = 0.023, η^2^ = 0.08]. A *post hoc* Tukey test revealed that this was due to longer MTs in the haloperidol versus the LD groups [*p* = 0.020]. Therefore, although we observed a significant drug effect on RT and MT during retention, this was consistent across reward and punishment.

### Feedback and drug status did not influence online error-reduction during visuomotor adaptation

Figure 2*A* shows the AD across epochs in the six groups. All groups showed clear error-reduction in response to the visuomotor perturbation with a main effect of block [*F*_(1.1,101.8)_ = 708.9, *p* < 0.001, η^2^ = 0.89, Greenhouse–Geisser corrected]. However, contrary to our expectations, this was not differentially affected by punishment versus reward [*F*_(1,90)_ = 1.69, *p* = 0.196, η^2^ = 0.018], or by drug status [*F*_(2,90)_ = 0.69, *p* = 0.505, η^2^ = 0.015].

### Reward enhanced retention but was not affected by LD or haloperidol

In the retention phase, we found a main effect of block [retention: *F*_(1.8,159.7)_ = 507.9, *p* < 0.001, η^2^ = 0.849, Huynh–Feldt corrected] suggesting participants gradually returned toward baseline performance (Fig. 2*A*). As predicted, there was a main effect of feedback [*F*_(1,90)_ = 9.8, *p* = 0.002, η^2^ = 0.098] with reward leading to greater retention than punishment (AD across retention phase, mean ± SEM -19.28 ± 0.62: R-Pl; -19.17 ± 0.93: R-LD; -16.87 ± 1.02: R-Halo; -16.65 ± 1.08: P-Halo; -14.38 ± 1.29: P-Pl; -16.14 ± 1.28: P-LD; Fig. 2*B*,*C*). However, drug status had no effect on retention [*F*_(2,90)_ = 0.44, *p* = 0.643, η^2^ = 0.010]. Although there was a significant block*feedback interaction [*F*_(1.8,159.7)_ = 3.29, *p* = 0.045, η^2^ = 0.035, Huynh–Feldt corrected], the lack of a block*drug [*F*_(1.8,159.7)_ = 2.34, *p* = 0.064, η^2^ = 0.050, Huynh–Feldt corrected], feedback*drug [*F*_(2,90)_ = 2.46, *p* = 0.091, η^2^ = 0.052] or block*feedback*drug [*F*_(1.8,159.7)_ = 1.73, *p* = 0.153, η^2^ = 0.037, Huynh–Feldt corrected] interaction suggests the effect of feedback was independent of drug status.

These results did not change when average MTs and RTs during retention were added as covariates; specifically there still was a nonsignificant effect of drug [MANOVA: *F*_(1,84)_ = 0.51, *p* = 0.602, η^2^ = 0.011]. In addition, a power analysis (G*Power 3.1.9.2) revealed our sample size gave us 91% power (1-β) to detect a significant block*feedback*drug interaction effect (*n* = 96, η^2^ = 0.037, effect size f = 0.196). This suggests that the nonsignificant effect of drug status on retention was unlikely due to an insufficient sample size, or drug-related differences in RT and MT.

### Model-based analysis confirmed model-free results

To estimate learning and retention rates from all available data, we also performed a model-based analysis by applying a single-rate SSM to each participant’s entire dataset (Thoroughman and Shadmehr, 2000; Donchin et al., 2003; Tanaka et al., 2009; Galea et al., 2015). The model was able to explain a substantial amount of variance (R^2^: 0.79, range 0.73–0.87: R-Pl, 0.80, 0.71–0.86: R-LD, 0.80, 0.68–0.87: R-Halo, 0.79, 0.71–0.85: P-Pl, 0.78, 0.66–0.88: P-LD, 0.80, 0.72–0.86: P-Halo), with a similar goodness of fit across groups [*F*_(5,90)_ = 0.62, *p* = 0.683, η^2^ = 0.033].

The SSM confirmed that error-reduction (learning parameter B, mean ± SEM 0.32 ± 0.04: R-Pl; 0.31 ± 0.03: R-LD; 0.32 ± 0.03: R-Halo; 0.34 ± 0.04: P-Halo; 0.41 ± 0.04: P-Pl; 0.40 ± 0.06: P-LD) was not differentially affected by punishment versus reward [ART test, *F*_(1,90)_ = 0.22, *p* = 0.639, η^2^ = 0.002], or by drug status [ART test, *F*_(2,90)_ = 0.19, *p* = 0.825, η^2^ = 0.004], with no significant feedback*drug interaction [ART test, *F*_(2,90)_ = 1.22, *p* = 0.301, η^2^ = 0.026]. In addition, there was no correlation across participants between executive functions (FAB, Stroop time, and Stroop error scores) and the learning parameter B (FAB: Spearman rho, ρ = 0.083, *p* = 0.422; Stroop time: ρ = -0.110, *p* = 0.298; Stroop errors: ρ = -0.034, *p* = 0.750).

Retention, represented by the decay parameter A (mean ± SEM 0.96 ± 0.002: R-Pl; 0.96 ± 0.003: R-LD; 0.95 ± 0.02: R-Halo; 0.95 ± 0.005: P-Halo; 0.94 ± 0.008: P-Pl; 0.94 ± 0.01: P-LD), was not affected by drug status [ART test, *F*_(2,90)_ = 1.08, *p* = 0.344, η^2^ = 0.023] but was influenced by feedback [ART test, *F*_(1,90)_ = 5.51, *p* = 0.021, η^2^ = 0.058], with reward leading to greater retention than punishment (Fig. 2*B*). The interaction between feedback*drug status was also not significant [ART test, *F*_(2,90)_ = 1.53, *p* = 0.223, η^2^ = 0.033]. Similarly to the learning parameter B, the decay parameter A was also not correlated with executive functions scores (FAB: Spearman rho, ρ = -0.101, *p* = 0.327; Stroop time: ρ = 0.040, *p* = 0.704; Stroop errors: ρ = 0.074, *p* = 0.488).

In summary, we showed that reward caused greater retention of the newly acquired motor memory relative to punishment. However, LD and haloperidol had no effect on either error-reduction or retention.

## Discussion

The aim of this study was to investigate the role of DA during a visuomotor adaptation task under reward or punishment conditions. Although we showed that reward-based feedback enhanced motor memory retention relative to punishment, this was unaffected by dopaminergic medication that either increased (LD) or decreased (haloperidol) DA availability in the brain.

### Reward led to higher memory retention than punishment

We found that reward-based feedback delivered during adaptation led to a greater amount of motor memory retention. This is in line with previous research in both healthy participants (Wächter et al., 2009; Abe et al., 2011; Galea et al., 2015) and stroke patients (Quattrocchi et al., 2017). Specifically, reward has been associated with increased retention across multiple motor learning tasks, ranging from sequence learning (Wächter et al., 2009; Wilkinson et al., 2015), to skill learning (Abe et al., 2011), visuomotor adaptation (Shmuelof et al., 2012; Galea et al., 2015), and force-field adaptation (Quattrocchi et al., 2017). These reward-related effects have been mainly associated with frontostriatal brain areas most commonly associated with DA (Wächter et al., 2009; Dayan et al., 2014; Hamann et al., 2014).

In addition, dopaminergic neurons in the VTA increase their firing in response to the presentation of rewards and to conditioned stimuli predicting reward (Volman et al., 2013; Schultz, 2016). At the same time, dopaminergic neurons from the rostro-lateral VTA, and to a lesser extent from the rostro-medial substantia nigra, project to M1 (Hosp et al., 2011). In animals, the integrity of these projections is necessary for the retention of new motor skills (Hosp and Luft, 2013). As there is evidence to suggest a role of M1 in human motor memory retention (Muellbacher et al., 2002; Richardson et al., 2006; Hadipour-Niktarash et al., 2007; Galea and Celnik, 2009; Reis et al., 2009), it is possible that dopaminergic projections to M1 could provide an underlying mechanism for the positive effects of reward on motor memory retention. On this basis, our hypothesis was that reward would increase motor memory retention through dopaminergic mechanisms.

### LD and haloperidol had no effect on error-reduction or retention

Surprisingly, LD did not influence the effect of reward. LD, the most widely and effective treatment used in Parkinson’s disease (PD), is converted to DA in the brain. Although motor and some cognitive symptoms in PD are improved by LD, others, such as motor sequence learning and probabilistic reversal learning, appear to be worsened (Swainson et al., 2000; Cools et al., 2001; Feigin et al., 2003; Ghilardi et al., 2007; Graef et al., 2010; Kwak et al., 2010). This paradoxical effect has been explained by the “dopamine overdose hypothesis,” suggesting that the effect of dopaminergic therapy on a function is determined by the baseline DA levels in the brain regions mediating that function (Vaillancourt et al., 2013). Therefore, we reasoned that the lack of effect of LD could be due to the already optimal DA levels in young healthy participants, rather than to the noninvolvement of dopaminergic pathways. Consequently, we hypothesized that if reward increased retention through dopaminergic mechanisms then by antagonizing DA function we should observe a deterioration of this effect. However, contrary to this expectation, the D1/D2-antagonist haloperidol, similarly to LD, had no effect on any phase of the experiment and, in particular, it did not decrease the effect of reward on motor memory retention.

Various hypotheses, not necessarily excluding each other, could explain these results. First of all, the lack of significance could be due to a small sample size. However, as described previously, a power analysis revealed we achieved 0.91 power to detect a significant block*feedback*drug interaction effect, thus suggesting that the nonsignificant effect of drug status on retention was not simply due to an insufficient sample size.

Secondly, it could be that the doses of LD and/or haloperidol used here were too low to have a behavioral effect. Indeed, previous evidence has suggested a dose-response effect of LD in regard to learning enhancement (Knecht et al., 2004). However, the oral doses used here have previously been employed in a range of studies, demonstrating clear behavioral and neurophysiological effects for both LD and haloperidol (Knecht et al., 2004; Pleger et al., 2009; de Vries et al., 2010; Adam et al., 2013; Bestmann et al., 2015). Despite this, as we did not observe any consistent global drug effect on behavior, it is possible that the doses used here were not sufficient to modulate the dopaminergic system. To overcome this possibility, future studies should investigate at least two tasks: an “experimental” one and another in which a consistent drug effect has already been demonstrated.

Additionally the between-subjects pharmacological approach, despite the advantage of directly manipulating the dopaminergic system, is nonspecific, and the administered drugs have widespread effects (Crockett and Fehr, 2014). In particular, it is well known that haloperidol acts at all levels of the central nervous system, primarily at subcortical levels, and that it also has strong antiadrenergic and weaker peripheral anticholinergic activity. Therefore, strictly speaking, our approach did not examine selectively just the dopaminergic pathways, and more studies are needed to directly and specifically investigate the dopaminergic circuitry in motor learning. Moreover, the genetic variability of DA receptors and DA cleaving or metabolizing enzymes could influence the effect of exogenous dopaminergic stimulation (Pearson-Fuhrhop et al., 2013). This confound could have been ruled out by using a within-subjects design, however this is not advisable in motor learning tasks as it introduces the problem of powerful carry-over effects (Crockett and Fehr, 2014; Huberdeau et al., 2015a). Finally, as all participants in this study received a tablet (either a placebo or an active drug), a placebo effect on retention and error-reduction in the placebo groups cannot be ruled out. Future work might wish to include a group in which no tablet is provided to discount this possibility.

Finally, it could be that the effect of reward on motor memory retention observed here is not DA dependent. On this point, we have to highlight that the current adaptation task does not disentangle the differential effects of positive or negative reinforcement on the multiple learning processes now known to influence performance (Smith et al., 2006; Taylor et al., 2014; Bond and Taylor, 2015; Huberdeau et al., 2015b; McDougle et al., 2015). For example, when participants made no vision movements we instructed them to “reach toward the target even without vision.” As this instruction was relatively ambiguous, the effect of reward on retention could either be due to participants maintaining the use of an explicit strategy or reflecting a highly stable reinforcement-based learning process (Smith et al., 2006; Taylor et al., 2014; Bond and Taylor, 2015; Huberdeau et al., 2015b; McDougle et al., 2015). Although the role of DA in reinforcement-based mechanisms is well known (Schultz, 2013), its importance for other cognitive processes is less clear. For example, Anguera et al. (2010) showed that visuomotor adaptation performance was correlated with a participant’s mental rotation working memory capacity. Interestingly, LD medication does not seem to improve PD patient’s ability to perform a mental rotation working memory task (Crucian et al., 2014). Therefore, it is possible that the positive effects of reward on motor memory retention are dependent on a cognitive (“frontal”) process unaffected by DA.

### Punishment showed no effect on error-reduction during visuomotor adaptation

Contrary to previous findings (Galea et al., 2015), we found no benefit of punishment on error-reduction in response to the perturbation. In both studies, we used a visuomotor perturbation, but the magnitude of the perturbation was larger here than in our previous paper (40° vs 30° in Galea et al., 2015). As the degree of explicit awareness is known to increase as a function of perturbation size (Werner et al., 2015), error-reduction here may have involved a greater use of explicit strategies. With smaller perturbations, the motivational salience of punishment (Kahneman and Tversky, 1979; De Martino et al., 2010) may motivate participants to use a strategy (and thus show faster error-reduction) in circumstances in which they are more difficult to develop. Conversely, in the present study punishment may have been unable to potentiate further an already well-represented explicit strategy. Therefore, we think that punishment may enhance performance during adaptation paradigms by increasing the use of a cognitive strategy, and that this becomes overtly beneficial in cases where this strategy is not yet optimally implemented. However, we are aware that this would not explain all the literature results (Song and Smiley-Oyen, 2017), and further examination of the effects of punishment on motor learning is clearly warranted. Additionally, the lack of effect makes it hard to evaluate the role of DA in motor learning under punishment.

### Implications and conclusions

This is the first direct pharmacological investigation on the role of DA in motor adaptation tasks under reward or punishment. Our results failed to support the hypothesis that reward increases motor retention through dopaminergic pathways. We here provide further evidence for a role of reward-feedback in adaptation tasks, but future work is needed to decompose the impact of reward on the various subprocesses involved in motor adaptation, and on the neural pathways underlining these mechanisms. In particular, this study highlights the critical role played by task instructions in investigating learning processes. In our specific case, for example, making subjects aware that the rotation was removed in the retention phase would have allowed us to decompose, and individually measure, the explicit component (disengaged by such explicit instructions) from the implicit one (Werner et al., 2015). Alternatively, we could have restricted the expression of explicit strategies through the use of a force-RT paradigm (Haith et al., 2015). Although we suggest that reward could be acting on the explicit component, there is also evidence that reward can modulate implicit adaptation processes (Kojima and Soetedio, 2017). Therefore, how reward and dopaminergic pharmacological manipulation influences the explicit and implicit components of adaptation is an exciting question for future research.

## Synthesis

Reviewing Editor: Trevor Drew, University of Montreal Decisions are customarily a result of the Reviewing Editor and the peer reviewers coming together and discussing their recommendations until a consensus is reached. When revisions are invited, a fact-based synthesis statement explaining their decision and outlining what is needed to prepare a revision will be listed below. The following reviewer(s) agreed to reveal their identity: John Morehead.

Reviewer 1

This is an interesting and timely studying investigating the mechanism underlying reward-based modulation of visuomotor learning and retention. The authors test the hypothesis that reward-based effects are mediated via dopaminergic mechanisms by administrating levodopa (dopamine precursor) or haloperidol (dopamine antagonist) to healthy adult subjects. Overall it is a null finding - the drugs did not affect learning or retention, suggesting that reward-based effects on learning are mediated via alternate mechanisms.

This is an important paper, given the recent surge of interest in non-error based factors in motor learning. Although this is a null result, it plays an important step toward understanding the neural mechanisms underlying reward-based modulation of motor learning. It provides valuable information upon which future studies can build upon, whether it be altering the dose, the type of drug, the subject population, or controlling for certain aspects of the task To the best of my understanding, the authors have appropriately controlled for confounding factors and the results will be of interest and of use to the field.

General comments

1. My one concern is with the placebo group and whether there may have been a strong placebo effect that confounded the results. The authors should clarify what instructions were given to the subjects upon taking the drug. What were they told they were taking? It is possible that a varying placebo response across subjects may have enhanced performance, or at least muddled the results in the Reward group. Similarly, variability in the response of the placebo group may have led to variable results in the Punishment groups.

Can the authors compare these results to a control group that receives reward feedback but no drug or placebo, perhaps from an earlier paper? It would at least be interesting, albeit not critical to this paper's conclusions, to see whether simply administering a pill can lead to improvements in retention, and it would more clearly implicate more ‘frontal’ processes.

2. The author's claim in the Discussion that they observe behavioral effects of the drugs on RT and MT. I suggest they remove this statement as their results are not strong. Difference are only found occasionally (in baseline and retention) , and would likely not survive corrections for multiple comparisons. Further, the placebo data is not consistent with this effect. It is sometimes even faster than the LD group. For there to be an effect - the proper comparison to be made is to placebo (as is shown in the Rammsayer paper cited).

3. Related to the point above, it would strengthen the paper if the authors could confirm some effect of the drugs that are similar to those observed in previous studies. The authors claim that they use the same dosage as other studies that have observed behavioral effects. Do the authors see anything similar in the present study? And if not, why?

Reviewer 2

Reward and punishment are hot topics in the fields of motor learning and sensorimotor adaptation. This work shows that previously reported dissociations in the effects of reward and punishment are not as reliable as we'd come to think. It also shows that pharmacological manipulations have a modest effect, and that young participants may not be affected by them much at all.

In this study, the authors sought to pharmacologically manipulate memories acquired in a visuomotor adaptation task. Specifically, they wanted to assess the roles and interactions of reward and punishment with haloperidol and levodopa in this learning context. The data is presented in a clear and straightforward manner, showing primarily that rewarding performance leads to less decay of the learned behavior. Unfortunately, there isn't much going on with the drug results. The authors do a nice job of discussing why there wasn't much of a drug effect, discussing limitations of their study, and directions for future work. I think there is valuable information here for the field. The discussion of the retention results alone brings up very important issues for our field, and I look forward to more people becoming aware that the instructions during this period can have such serious consequences.

Some additional comments:

I think the discussion (maybe in the implications part?) should include a section that talks about possible solutions to the problem that arose from the instructions for the retention block (discussed in lines 419-451). How can this issue be avoided in the future? Do we always need to run two groups, a ‘maintain’ and ‘stop’ group? Do we always need to measure aim? Are there other techniques that would shed more light on this? Would any of these be more or less sensitive to pharmacological manipulations?

I think Figure 2 could be amended to show the individual data for each bar graph. This is becoming more and more the style of presentation for data, and I think it would be a good thing here.

I think the r squared values for the state-space fits should be reported. Not every single one, but maybe the ranges. More importantly, it might not be a good idea to use parameters from fits that aren't very good.

There's a typo on line 24, ‘brain Therefore’ needs a period.
